# Consumption of barracuda in the Caribbean Sea linked to ciguatera fish poisoning among Filipino seafarers

**DOI:** 10.5365/wpsar.2016.7.2.004

**Published:** 2018-11-13

**Authors:** Niño Rebato, Vikki Carr de los Reyes, Ma Nemia Sucaldito, Flor D’Lynn Gallardo, Julius Erving Ballera, Irma Asuncion, Kenneth Hartigan-Go

**Affiliations:** a FETP-Philippines, Philippines.; b Department of Health, Philippines.

## Abstract

**Introduction:**

Ciguatera fish poisoning (CFP) is common in tropical and subtropical waters. On 13 November 2015, eight Filipino seafarers from a cargo ship sailing in the Caribbean Sea experienced a range of symptoms after consuming a barracuda. Upon their return to the Philippines, an investigation was conducted to describe the cases.

**Methods:**

A case-series was conducted. A CFP case was defined as a previously well individual on the ship who developed at least one gastrointestinal symptom and at least one neurologic manifestation after eating barracuda on 13 November 2015. All cases were admitted to hospital in Manila, Philippines and were interviewed using a standard questionnaire. Urine and serum samples of cases were collected for ciguatoxin (CTX) testing by radiological and receptor-binding assay.

**Results:**

Eight of the 25 seafarers on the ship ate the barracuda; all eight met the CFP case definition. The age of cases ranged from 37 to 58 years (median: 47 years) and all were males. Onset of symptoms ranged from 1 to 3 hours (median: 2 hours) from the time of ingestion of the barracuda. All cases experienced gastrointestinal (nausea, vomiting, diarrhoea) and neurologic (temperature allodynia, itchiness) symptoms but no cardiovascular manifestations. Urine and serum specimens of all eight cases showed CTX below the detection limit.

**Discussion:**

The Philippines Epidemiology Bureau recommended that the Philippine Maritime Authority include CTX poisoning and its health risks in seafarers’ training to prevent future cases of CFP. The Event-based Surveillance and Response system will continue to provide a mechanism for the reporting and appropriate management of CFP cases.

## Introduction

Ciguatera fish poisoning (CFP) is widespread in tropical and subtropical waters (*1*) and is acquired from consuming contaminated reef fish. The ciguatoxin (CTX) comes from the dinoflagellate *Gambierdiscus toxicus,* which grows predominantly in coral reefs in tropical and subtropical climates (*2*) and is consumed by herbivorous fish, which in turn are consumed by carnivorous reef fish and then by humans. (*3*) There are several reefs where fish such as barracuda and grouper are inedible because of the toxin; however, the toxin does not affect all reef fish, and deep-sea fish such as tuna and wahoo are unaffected. (*4*) Temperature, gastric acid and cooking method have no effect on the ciguatoxin, and its presence does not affect the odor, colour or taste of the fish. (*5*)

CFP is diagnosed clinically based on a cluster of symptoms temporally related to the ingestion of suspected fish products. All people can be affected by this toxin, and symptoms may persist for months or years. (*6*) Neurologic symptoms usually last for a few days to several weeks and may sporadically reoccur years later. (*7*) Triggers for reoccurrence may include consuming seafood, chicken, nuts, caffeine or alcohol and strenuous physical activity. (*8*)

CFP among seafarers is rarely documented, and treatment is usually delayed because they are at sea without medical facilities. Previously in the Philippines, an outbreak of CFP was documented in 2001 among 38 residents of Navotas who ate barracuda caught in Manila Bay; remnants of the implicated fish tested positive for CTX. (*9*) In 2010, two families with 22 members experienced gastrointestinal and neurologic symptoms after eating red snapper caught by a local fisherman in Iloilo, Philippines; it also subsequently tested positive for CTX. (*10*)

On 29 November 2015, the Epidemiology Bureau of the Philippines received a report of suspected CFP among seafarers who had consumed barracuda on 13 November 2015 in St Eustatius, a Dutch island in the Caribbean. Cases had been hospitalized in Trinidad and Tobago, then repatriated back to the Philippines and readmitted to a medical centre in Manila. An epidemiologic investigation was conducted to describe the cases.

## Methods

A descriptive study was conducted on 3 December 2015 while seafarers were still hospitalized in Manila. The cases were identified by the surveillance officer of the hospital from the list of admitted cases. A CFP case was defined as a previously well individual who developed at least one combination of gastrointestinal (diarrhoea, abdominal pain, nausea or vomiting) and neurologic manifestations (dizziness, weakness and itching or temperature allodynia) after eating barracuda on the cargo ship on 13 November 2015. Cases were interviewed to collect demographic and clinical information, food intake history and food preparation of barracuda using a standard questionnaire with both open- and close-ended questions developed by the investigators.

Urine and serum samples of cases were collected and submitted to the Philippines Nuclear Research Institute to detect CTX using receptor-binding assay (RBA). (*11*)

All data analyses were conducted using Microsoft Excel 2013. Ethical clearance was waived as this investigation was part of a response to an outbreak.

## Results

**Fig. 1:**
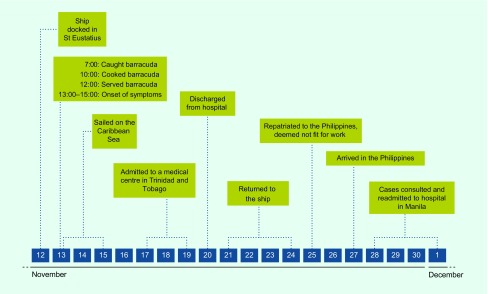
Timeline of events, CFP outbreak among Filipino seafarers who returned from St Eustatius, Kingdom of the Netherlands, 2015

There were 25 seafarers on board the ship, with eight having lunch at 12:00 on 13 November 2015. All of the cases reported consuming the barracuda, with smaller numbers also consuming rice (*n* = 7), egg (*n* = 4), ham (*n* = 4) and chicken (*n* = 2). After 1 to 3 hours (median: 2 hours), all eight cases manifested symptoms of nausea, vomiting, diarrhoea, itchiness and temperature allodynia (reversal of thermal sensation) (**Fig. 1**). The age of cases ranged from 37 to 58 years (median: 47 years) and all were males. Cases were given liquid charcoal as first aid for food poisoning and antispasmodic medicine to help relieve diarrhoea. There was no cardiovascular manifestation observed. Patients had no previous history of CTX poisoning. There was also no history of alcohol consumption within the week leading up to the event.

Urine and serum samples were collected from all eight cases, but the toxin was not detected.

## Discussion

Eight cases of CFP were reported after consumption of barracuda in the Caribbean Sea. All seafarers who consumed the same barracuda experienced gastrointestinal and neurological signs and symptoms typical of CTX. (*10*, *12*) None of the cases had cardiovascular complications, which are observed in about 10–15% of CTX cases. (*13*) In humans, the average dose of CTX required to develop symptoms is estimated to be as low as 0.08 to 0.1 µg/kg of the body weight. (*8*)

Rising ocean temperatures can affect wind patterns, which can force warm tropical waters to non-CFP endemic coastal regions. (*14*) As a result, dinoflagellates producing ciguatoxins have expanded their presence to previously non-CTX affected oceans, increasing risk of CFP among those consuming fish from these oceans. CFP causes significant impact on marine operations because of the high attack rates and chronicity of symptoms, which can result in seafarers requiring long periods of recovery. Providing information about potential marine products that can be contaminated with CTX to this high-risk group may help mitigate the occurrence of CFP.

There were several limitations of this study. The outbreak occurred while the cases were sailing in the Caribbean Sea; hence, no leftover fish was available for toxin analysis and bacterial culture. The species of the fish was not confirmed; rather, the seafarers who caught the fish identified it as barracuda. Also, we were not able to interview the person who caught and prepared the food for the cases; hence, the food production chain could not be established and investigated. It is known that larger fish in the food-chain have higher levels of accumulated toxins, (*15*) but no information on the actual size of the fish caught was elicited. That CTX was not detected could be attributed to the extremely low levels (*11*) and fast α half-life of the toxin, leaving undetectable concentrations of the toxin in the blood. (*16*) Bacterial culture was not done to test for other pathogens because the time course, symptoms, food-specific attack rate and history of eating the fish strongly supported the CFP diagnosis. (*8*)

The Epidemiology Bureau recommended that the Philippine Maritime Authority include CTX poisoning and its health risks in the seafarers’ training to prevent future cases of CFP. CTX continues to be reportable in the Event-based Surveillance and Response system of the Department of Health. Coordination with the Bureau of Fisheries, Philippine Nuclear Research Institute and Department of Health will enable immediate detection of CFP for appropriate management of cases, reducing serious implications of CFP.

## References

[1] Pottier I, Vernoux JP, Lewis RJ. Ciguatera fish poisoning in the Caribbean islands and Western Atlantic. Rev Environ Contam Toxicol. 2001;168:99–141. 10.1007/978-1-4613-0143-1_3 12882228

[2] Nellis D, Barnard G. Ciguatera: a legal and social overview. Mar Fish Rev. 1986;48(4):2–5. https://spo.nmfs.noaa.gov/mfr484/mfr4842.pdf

[3] Babinchak JA, Jollow DJ, Voegtline MS, Higerd TB. Toxin production by ***Gambierdiscus toxicus*** isolated from the Florida Keys. Mar Fish Rev. 1986;48(4):53–6. https://spo.nmfs.noaa.gov/sites/default/files/pdf-content/MFR/mfr484/mfr48412.pdf

[4] Centers for Disease Control and Prevention (CDC). Ciguatera fish poisoning–Texas, 1997. MMWR Morb Mortal Wkly Rep. 1998 8 28;47(33):692–4. 9733416

[5] Shibamoto T, Bjeldanes L. Introduction to food toxicology. 2nd ed. Cambridge (MA): Academic Press; 2009.

[6] Lehane L, Lewis RJ. Ciguatera: recent advances but the risk remains. Int J Food Microbiol. 2000 11 1;61(2-3):91–125. 10.1016/S0168-1605(00)00382-2 11078162

[7] Miller DM. Ciguatera seafood toxins. 1st ed. Boca Raton (FL): CRC Press; 1990.

[8] Friedman MA, Fleming LE, Fernandez M, Bienfang P, Schrank K, Dickey R, et al. Ciguatera fish poisoning: treatment, prevention and management. Mar Drugs. 2008;6(3):456–79. 10.3390/md6030456 19005579 PMC2579736

[9] Tante S. Ciguatera fish poisoning outbreak in Navotas, Metro Manila. Manila: Epidemiology Bureau Library; 2001.

[10] Mendoza CO, Rabanes AC, Jimenez EC, Azanza RV, Cortez-Akhunzadah J, Cruz LJ. Detection of ciguatera fish poisoning in the Philippines. J Environ Sci Manag. 2013 1;16(1-2013):50–5.

[11] Detection of harmful algal toxins using the radioligand receptor binding assay. A manual of methods. Vienna: International Atomic Energy Agency; 2013 (https://www-pub.iaea.org/MTCD/Publications/PDF/TE-1729_web.pdf, accessed 25 September 2018).

[12] Schlaich C, Hagelstein JG, Burchard GD, Schmiedel S. Outbreak of ciguatera fish poisoning on a cargo ship in the port of hamburg. J Travel Med. 2012 7;19(4):238–42. 10.1111/j.1708-8305.2012.00619.x 22776385

[13] Senthilkumaran S, Meenakshisundaram R, Michaels AD, Suresh P, Thirumalaikolundusubramanian P. Cardiovascular complications in ciguatera fish poisoning: a wake-up call. Heart Views. 2011 10;12(4):166–8. 10.4103/1995-705X.90905 22574244 PMC3345153

[14] Heimann K, Capper A, Sparrow L. Ocean surface warming: impact on toxic benthic dinoflagellates causing ciguatera. Hoboken (NJ): John Wiley & Sons, Ltd; 2011. [cited 2017 March 31]. Available from: http://www.els.net/WileyCDA/ElsArticle/refId-a0023373.html

[15] Arnold TC, Tarabar A. Ciguatera toxicity. New York (NY): Medscape; 2015. [cited 2015 December 4]. Available from: http://emedicine.medscape.com/article/813869-overview

[16] Ledreux A, Ramsdell JS. Bioavailability and intravenous toxicokinetic parameters for Pacific ciguatoxin P-CTX-1 in rats. Toxicon. 2013 3 15;64:81–6. 10.1016/j.toxicon.2012.12.026 23319077

